# Toward Transparency on Animal Experimentation in Switzerland: Seven Recommendations for the Provision of Public Information in Swiss Law

**DOI:** 10.3390/ani14152154

**Published:** 2024-07-24

**Authors:** Nicole Lüthi, Christian Rodriguez Perez, Kirsten Persson, Bernice Simone Elger, David Shaw

**Affiliations:** 1 Faculty of Law, University of Zurich, 8032 Zurich, Switzerland; 2 Institute for Biomedical Ethics, University of Basel, 4056 Basel, Switzerlandb.elger@unibas.ch (B.S.E.);; 3 Institute for Animal Hygiene, Animal Welfare and Farm Animal Behaviour, University of Veterinary Medicine Hannover, 30173 Hannover, Germany; 4 Center of Legal Medicine, Faculty of Medicine, University of Geneva, 1000 Geneva, Switzerland; 5 Care and Public Health Research Institute, Maastricht University, 6229 Maastricht, The Netherlands

**Keywords:** transparency, animal experimentation, Swiss law, animal welfare law, surplus animals

## Abstract

**Simple Summary:**

Simple Summary: In Switzerland, the importance of transparency in animal experimentation is emphasized by the Swiss Federal Council, in recognition of the public’s great interest in this matter. Current Swiss law requires institutions and the government to inform the public about various aspects, such as the number of animals used in experiments, their species, or the severity of harm of the experiment. However, much relevant information is missing, such as the number of breeding or surplus animals, the fate of the animals (both in facilities and after an experiment), and how the balancing of animal harm and human benefits has been performed to justify a particular experiment. Considering that the Swiss government has a duty to provide information on animal experimentation conducted on the public’s behalf, such information should be disclosed. If Switzerland is to move toward more transparency in public information on animal experimentation, an update of the legal requirements is needed. In this article, we give recommendations for Swiss law to move toward more transparency in public information.

**Abstract:**

In Switzerland, the importance of transparency in animal experimentation is emphasized by the Swiss Federal Council, recognizing the public’s great interest in this matter. Federal reporting on animal experimentation indicates a total of 585,991 animals used in experiments in Switzerland in 2022. By Swiss law, the report enables the public to learn about many aspects such as the species and degree of suffering experienced by the animals, but some information of interest to the public is missing, such as the fate of the animals at the end of the experiment (e.g., euthanized, rehomed in a private home, reused in another experiment). When it comes to animals bred in facilities but not used in experiments, further information of interest is not required to be made public according to Swiss law, for example, the number and fate of “surplus” animals (i.e., animals bred but not used in experiments for a variety of reasons such as not carrying the phenotypical properties needed). Considering that the Swiss government has a duty to provide a full accounting of animal experimentation conducted on the public’s behalf, further relevant information should be disclosed. While efforts toward transparency, such as the STAAR Agreement, have been made in the scientific community, these mostly reflect the legal requirements already in force. If Switzerland is to move toward more transparency in public information on animal experimentation, an update of the legal requirements is needed. In this article, we give recommendations for Swiss law to move toward more transparency in public information on seven aspects: (1) the fate of the animals at the end of the experiment; (2) the sources of funding for animal experimentation; (3) the harm-benefit analysis performed by researchers and ethics committees to justify an experiment using animals; (4) the number of breeding/surplus animals; (5) the fate of breeding/surplus animals; (6) the harms experienced by animals in facilities; and (7) the funding of animal facilities.

## 1. Introduction

Swiss law requires researchers and animal facilities to both document and report different types of data. In 2022, Switzerland reported a total of 585,991 animals used in experiments. The public can learn about the species of and degree of harm experienced by these animals (Art. 24 and 25 of the Animal Experimentation Ordinance (AEO) define four degrees of severity of harm in the context of animal research, which include severity 0–3, where: 0 indicates no harm; 1 indicates mild harm; 2 indicates moderate harm; and 3 indicates severe harm), as well as the areas of experimentation they are used for in the annual report. Moreover, it shows that a total of 1,262,383 animals were born or imported into Swiss animal facilities. While these data provide answers to important ethical questions of interest to the public (e.g., How many animals experienced experiments of the maximal severity of harm? Which types of species have been used the most? What are the trends when comparing the numbers to previous years?) they fail to address crucial others.

Political initiatives, both in Switzerland [1] and in the European Union [2,3] show that the public cares about all types of animal use in experimentation. Knowing the number of animals used in experiments is important. Still, the lack of data on the experience of “breeding animals” (i.e., animals used in facilities for breeding only) and so-called “surplus animals” (i.e., animals bred for experimentation but not used in experiments for a variety of reasons, such as not carrying the phenotypical properties needed) conveys the idea that their lives (and deaths) involve no harm whatsoever. While the term “surplus animals” is widely used in the literature, it does not appear in Swiss or European law. Swiss and EU reports rather talk about animals “bred but not used” (CH stat report) or “bred, killed and not used in procedures” (EU stat report). In this paper, we use the term “breeding/surplus animals” to refer to all animals involved in animal experimentation but not used in actual experiments.

Most certainly, the public cares about the killing of animals [4]. But again, Swiss citizens currently have no way of knowing how many animals are killed for science, whether it is after having been used in an experiment or as a breeding/surplus animal. Finally, although the Swiss Constitution protects the dignity of all creatures (Art. 120 para. 2 Cst.), the animals considered in this context are only a subgroup, i.e., those exclusively protected under Swiss *animal experimentation law* (i.e., vertebrates; cephalopods; decapods; mammals, birds, and reptiles in the last third of the gestation period before birth or hatching; larval stages of fish and amphibians that take in food autonomously (Art. 112 AniPO)—we refer to these when using the term “animal” in this paper. Given the growing scientific evidence of the sentience of animals that are currently not protected by Swiss law [5], the question may arise as to how transparent the state should be about its use of these animals in experimentation (e.g., drosophila). While we support the prompt and scientifically sound inclusion of all sentient species in animal welfare legislation, for reasons of complexity and feasibility, we will focus solely on animals protected under Swiss law.

Below, we will argue that this lack of transparency is ethically and legally problematic. While efforts to increase transparency, such as the STAAR Agreement (i.e., a coalition of Swiss universities and other institutions aiming at more transparent communication with the public about animal experimentation) [6] have been made in the scientific community, these mostly reflect the legal requirements already in force. If Switzerland is to move toward more transparency in public information on animal experimentation, an update of the legal requirements is needed. In this article, we give recommendations for Swiss law to move toward more transparency in public information. After defining our our account of transparency, we analyze current Swiss law on the issue and continue by comparing it to the general European, and in particular the German context. Finally, we give recommendations for increasing the transparency required by Swiss law on seven aspects: (1) the fate of the animals at the end of the experiment; (2) the funding of animal experimentation; (3) the harm-benefit analysis performed by researchers and ethics committees to justify an animal experiment; (4) the number of breeding/surplus animals; (5) the fate of breeding/surplus animals; (6) the harms experienced by animals in facilities; and (7) the funding of animal facilities.

## 2. What Is Transparency and Why Is It Important?

Transparency can have many different meanings. As a result, there is no single definition of the term ‘transparency’. However, it is often commonly understood in relation to “publicness”, “openness”, “disclosure”, “accountability”, or “awareness” [7]. Among its many functions and values, transparency in particular has become synonymous with “democracy” [8]. In what follows, we will therefore briefly discuss the meaning and importance of transparency in democracy to provide a more general overview of the concept, before turning to animal experimentation and specifying the definition of transparency we use in this article. We will do so with a particular focus on the Swiss context.

### 2.1. Transparency in Democracy

In the legal sense, transparency is often equated with the “accessibility of government information” and is a central precondition for the rule of law and democracy [9,10,11,12]. In this context, it is argued that “transparency is a *conditio sine qua non* for the *informed* consent of the governed” or more simplified “democracy needs transparency” [7]. Transparency makes it possible to monitor the actions of governments, thereby strengthening the legitimacy of democracy [7,12,13]. It is also a key instrument to ensure informed and equal participation of citizens in a democracy [11,12,13,14]. Only well-informed citizens are in a position to form their own opinions and take an active part in the decision-making process of society and the state [7,11,15,16]. Furthermore, transparency prevents asymmetries in knowledge and power, which in turn builds trust [13].

In line with this, Brian Kogelmann shows that political philosophers tend to argue that the consent of the majority is a necessary condition of legitimate democratic state action, except in rare specific cases in which secrecy is needed (e.g., secret military action) [17]. In this sense, laws in democratic states must be consented to by the majority in order to be legitimate. Since consent is not possible without transparency, transparency is also a necessary condition of legitimate action. Transparency can therefore be seen as a driver for public participation and consensus building [7]. A lack of transparency in a democracy can lead to the formation of special interest groups and distort democratic processes, as those who are not informed are not encouraged to participate actively [7].

In the Swiss context, in terms of Swiss public law, the principle of transparency is to be understood as an “unwritten principle of constitutional law” [11,13,18]. In this sense, it goes beyond the principle of public access within the meaning of Art. 6 para. 1 of the Freedom of Information Act (FolA). Thus, the principle of transparency applies not only to the accessibility of official documents but also to the actions of the state as a whole. This includes all government processes [13]. It is also not enough to simply make information about government processes public. Rather, the principle of transparency requires that information also be made available to the public in a comprehensible manner [11]. Consequently, to ensure a functioning and equal democracy, a constant, comprehensible and transparent communication between a state and its society is essential. It is therefore the state’s duty to ensure that the principle of transparency is respected [13].

Here again, some political philosophers emphasize the particular relationship between democratic governments and the public. For example, the philosopher Jeremy Waldron considers this relationship key and argues that it should be understood in terms of *agent accountability,* as follows:

*Agent accountability*: In this conception, “accountability” denotes the duty owed by an agent to his principal, whereby the principal may demand from the agent an account of the work that the agent has been doing in the principal’s name or on the principal’s behalf, enabling the principal if she sees fit to sanction or replace the agent or terminate the agency relationship [19].

In his view, the democratic government should be considered an agent that has a duty to the public. What is particular about this view, in contrast with narrower definitions of accountability, is that the public itself decides the basis on which it will assess the government’s actions [19]. Waldron also states that it is not the public’s role to come up with ways of keeping track of what its government is doing, but it is rather the government’s duty to generate that transparency and convey the information that accountability requires [19]. In this sense, citizens are entitled to a full account of what their government does in their name or on their behalf, in particular about the aspects that matter most to them, enabling them to evaluate, sanction, or even terminate the relationship [17]. It is on this view and on the notion of full accountability, to which the public is entitled and which is the government’s duty, that we will focus our definition of transparent public information.

### 2.2. Transparency in Animal Experimentation

As we have seen, transparency has many facets and is of particular importance in a democracy. But the question remains as to why transparency is also essential in animal experimentation. Before discussing this, however, it is important to note that there are many stakeholders involved in animal experimentation, each with a different understanding of transparency and different preferences regarding transparency in this context. Therefore, the meaning of the term “transparency” and the threshold of what constitutes adequate transparency may be interpreted differently by different groups, including researchers, animal welfare advocates, and the general public [20].

Consequently, different stakeholders will also have different reasons for believing that animal experimentation should be more transparent. For example, researchers may, on the one hand, argue that transparency is important to counteract potential damage to their public image and to increase public trust in the conduct of animal experimentation [21,22]. This is also shown by several initiatives of the scientific community in the past, e.g., the Basel Declaration [23] and more recently the STAAR Agreement [6]. However, both the Basel Declaration and the STAAR Agreement in large part reflect the legal requirements already in force concerning animal experimentation.

Animal welfare advocates, on the other hand, may argue that transparency is important to raise public awareness, stimulate public debate, ultimately improve animal welfare standards, and accelerate the use of alternative methods [22]. Here too, several initiatives have already been taken by animal welfare organizations in the past [24,25]. In addition, political initiatives [26,27,28] and public consultations show that transparency in animal experimentation is also important to the public. For example, surveys conducted in the EU between 2006 and 2023 revealed public demand for transparency in animal experimentation [29]. The public expressed interest in various key details, including information about the killing of laboratory animals, levels of suffering, or alternatives to animal use [30]. Another survey conducted by the European Commission in 2006 found that 90 percent of the respondents would like to see more transparency and even have a say in the question of when and how animal experiments may be carried out [31].

With this in mind, it seems only reasonable that people should be informed about the (financial and intangible) costs of animal experimentation since they benefit (at least to some extent) from animal experimentation in terms of knowledge gains, and even more directly when they take medicines or undergo certain treatments [32]. In some sense, if in journalism transparency has been presented as a way to confront the old “*we write, you read*” dogma [33], in animal experimentation it can address a “*we use* (animals), *you benefit*” dogma. This is all the more true as animal welfare is also a legitimate public interest and the use of animals in research is a (growing) ethical concern [34,35] for many people [32]. With a full account of the use of animals in research, citizens are enabled to make more informed decisions and contribute more actively to democratic life [32]. Only if citizens are properly informed can a consensus be reached on whether and under what conditions animal experimentation is acceptable and what ethical standards should be respected. Furthermore, a lot of experimentation is state-funded. In Switzerland, the estimated annual federal costs of animal experimentation amount to 46 million Swiss francs. In addition, the Swiss cantons spend approximately 25 million annually [36]. Thus, the argument has been brought forward that the public is entitled to know what impact their taxes and donations have on laboratory animals [32]. Greater transparency regarding the funding of animal experimentation would allow the public to draw conclusions about the efficiency of research [36].

In addition, it has been argued in the context of human research, that transparency is crucial to ensure public scrutiny and ethical standards [13]. This argument also applies to animal experimentation. With regard to the Swiss context, animals are recognized as sentient beings with intrinsic value, and their dignity is protected (Art. 120 para. 2 Cst; Art. 1 AniWA). Therefore, experiments that cause them harm can only be justified if the research generates a societal benefit [37]. In Swiss animal experimentation law, this is reflected in the “indispensable minimum” (Art. 17 AniWA) and the harm-benefit-analysis (Art. 19 para. 4 AniWA), requirements which (among other things) are intended to ensure that only high-quality animal experiments are carried out [38]. Non-disclosed research cannot be sufficiently legitimized with regard to the transparency principle, particularly in an ethically highly sensitive area such as animal experimentation. Referring to the International Monetary Fund and the World Bank, Economist and Nobel laureate Joseph E. Stiglitz pointed out that “public scrutiny will put a check on the most abusive practices” and that “it can increase the likelihood that the policies that are in the general interest—not just in the special interest of, say, the financial community—are pursued” [39]. The same holds for animal experimentation: public scrutiny is essential to ensure that researchers are being held accountable to high standards and requirements of ethical conduct. Moreover, just like in finances, public scrutiny in animal experimentation can increase the likelihood of a legal and ethical framework that reflects the public’s interest. This increased public scrutiny is even expected to lead to a decrease in the number of animals used in scientific research by some authors [20].

In summary, while there are diverse interpretations and rationales surrounding transparency in animal experimentation, there is a broad consensus on its importance. Although transparency in animal experimentation can and should include more than accountability (e.g., involvement of the public in the decision-making [40] and collaborations between the media and the scientific community [32]), this article will focus on public information and the particular duty of the government to provide disclosure. Therefore, the critical question remains as to what information should be disclosed to the public. In this sense, the question that interests us is: what information on animal experimentation should be part of the government’s full accounting to the public? While it is argued that reaching full transparency is impossible [40], a full account should aim to include all the information necessary for the public to assess the government’s animal experimentation activity done on its behalf. This means disclosing the ethically, politically, and legally relevant facts and the facts that matter to the public and enabling an informed public debate on animal experimentation. As stated above, the principle of transparency also requires that information is made available to the public in a comprehensible manner. In this sense, in addition to figuring out “what” the relevant facts are, governments have a duty to figure out “how” (e.g., text reports, figures, pictures, videos) and “where” (e.g., websites, printed documents, other media) to disclose them. While this article focuses mainly on making recommendations about “what” should be added to the Swiss government’s full account, we will briefly discuss the “how” and “where” below. However, we will first provide an overview of the current transparency requirements for the public in animal experimentation in Switzerland and the EU.

## 3. Transparency Requirements in Switzerland

Looking more closely at the Swiss context, the importance of transparency in animal experimentation is emphasized by the Swiss Federal Council [38], recognizing the public’s great interest in this matter. The Council states that “transparency in the sensitive area of animal experiments” needs to be improved [38]. It is the Swiss legislator intention (and duty) to create transparency to promote an objective public dialogue about animal experimentation. The Swiss Academy of Medical Sciences (SAMS) also highlights transparency in its ethical principles and guidelines for animal experiments [41]. Although the guidelines are not legally binding, they have considerable influence [42].

As the licensing and supervisory authority, the Swiss state must meet high standards of transparency in animal experimentation (Art. 18, 25 AniWA). In addition, high transparency requirements must apply all the more to state actors carrying out the majority of animal experiments, such as universities and cantonal hospitals [43].

### 3.1. Documentation and Reporting Requirements

In Switzerland, both the husbandry of laboratory animals and animal experiments require authorization through a licensing procedure (Art. 18 AniWA; Art. 122 Animal Protection Ordinance (AniPO)) to ensure compliance with the relevant requirements. To uphold these during experiments as well, animal welfare legislation imposes further documentation and reporting requirements for facilities and researchers. This not only facilitates supervision by the authorities but also collects the relevant data to inform the public about animal experimentation [44,45,46].

The main focus of the documentation and reporting requirements is the “animal inventory control”, mandatory for all laboratory animal facilities (Art. 18 para. 5 AniWA). Art. 143 para. 1 of AniPO specifies that the required documentation must contain information on the number of animals bred in the facility or imported from abroad as well as those leaving the facility (e.g., due to being transferred to another private or public facility or—more likely—due to death of the animal) within a year. If an animal has died, the cause of death must be documented if known (Art. 143 para. 1 lit. b AniPO). However, only the number of animals bred in the facility or imported from abroad per calendar year is subject to reporting requirements (Art. 145 para. 1 AniPO; Art. 29 para. 1 AEO). Accordingly, the number of animals that died or were transferred in laboratory animal facilities need only be documented and not reported. This is set to change with the ongoing revision of the Animal Welfare Ordinance and the AEO. In particular, an amendment to Art. 145 para. 1 of AniPO and Art. 29 para. 1 of AEO has been proposed. The number of animals bred, produced, and imported per calendar year as well as their fate after an experiment are to be included in the reporting obligation. This would include animals that are not used in animal experimentation or breeding and are killed (i.e., surplus animals), as well as animals that have died of natural causes in laboratory animal husbandry. In addition, a distinction is to be made between animals used for breeding and those used for experiments [47]. This planned revision aims to create transparency to reduce the number of animals bred for research purposes [48].

Special requirements apply to genetically modified animals (Art. 2 para. 3 lit. v AniPO) and “mutants”, i.e., animals that genetically experience pain or harm or suffer due to a stress-induced mutation that may be spontaneous, physically or chemically induced, or genetically engineered (Art. 2 para. 3 lit. k AniPO) For genetically modified animals, the “inventory control” must also contain a documentation of harm (Art. 124 AniPO; Art. 12 et seq. AEO), while lines or strains with mutants have to be reported (Art. 145 para. 1 lit. b and Art. 126 AniPO).

Not only laboratory animal facilities but also researchers have to comply with the documentation and reporting requirements for their activities (Art. 144 AniPO). According to Art. 145 para. 2 AniPO, they must report, for example, the final number of animals used in the experiment and the classification of the actual degree of severity of the experiment. It is noteworthy that the reporting requirements also include the fate of the animals after the experiment (Art. 31 para. 1 lit. e AEO).

Once the facilities and researchers have sent their data to the cantonal authorities, the latter forward them to the Federal Food Safety and Veterinary Office (FSVO) (Art. 145 para. 4 AniPO). This is accomplished using the electronic information system ‘Animex-ch’ [49], managed by the FSVO [46]. While the public does not have access to Animex-ch (cf. Art. 20c para. 1 AniWA), the FSVO uses it as a basis for public information about animal experimentation, which is described below.

### 3.2. Public Information

Art. 36 of AniWA obliges the FSVO to publish annual statistics showing all uses of laboratory animals [43]. According to Art. 147 para. 1 AniPO the statistics “must contain the necessary information to assess the application of animal welfare legislation in the areas of animal experimentation, laboratory animals and genetically modified animals.”

The current annual statistics break down the use of laboratory animals by species, purpose of use (e.g., basic research, education, or disease diagnostics), degree of severity, and the number of animals used in experiments in each Swiss canton [43]. Further information can be accessed as part of the extended statistics and compared with previous years. These include the category of institution in which the animal experiments were carried out (e.g., hospitals, universities, cantons, or private industries), the origin of the animals used, and the diseases that were studied in the experiment (e.g., cancer, cardiovascular diseases, or animal diseases) [43]. The statistics also show the number of authorized and declined animal experiment applications [43]. The data are summarized and commented upon in an accompanying report [43]. However, Swiss law does not specify exactly how and where this information should be published. Currently, the statistics and the accompanying report are only published online on the FSVO’s website, which may not be accessible to everyone. While the statistics and a summary of the annual report are provided directly on the website and are quite easy to access, the full report must be downloaded.

If an animal happens to be used in more than one research project in a year, each use is recorded in the statistics. Animals that have already been used in experiments in the previous year must be recorded each year and are also listed in the statistics [43]. In contrast to the European Union (EU), Switzerland does (currently) not include surplus animals in its publicly available animal experimentation statistics despite the law requiring these data to be recorded by facilities [50].

Since 2014, the FSVO has also published the reported data on animals kept in facilities (see above). Comparing the number of animals born in or imported into Swiss facilities with the number of animals actually used in experiments can give the public a sense of how many breeding/surplus animals exist in Switzerland. We said above that transparency requires making information comprehensible, but such calculations regarding the number of breeding/surplus animals are complex and it is unlikely that most citizens would engage in them. In any case, the actual number of breeding/surplus animals is not published [50,51,52,53,54].

In addition to the annual animal experimentation statistics, the FSVO publishes the experiments completed as defined in Art. 20a AniWA every quarter (cf. Art. 145a AniPO). In compliance with Art. 20a para. 1 of AniWA, information on the title, subject area, research objective, number of animals used per species, and degree of severity is published after the completion of every experiment. This information, however, is already included in the annual statistics. Moreover, critics have said that the presentation of the data under Art. 20a of AniWA is neither clear nor user-friendly [54]. Currently, this information is published on the FSVO website in several downloadable Microsoft Excel spreadsheets that have to be manually linked to each other, which undermines the validity—and transparency—of this information [26].

Thus, despite the legislature’s efforts to provide for more transparency by breaking down this information per experiment [38], it remains difficult for the public to gain an informed understanding from the published data of what exactly is being done to the experimental animals, under what conditions they are kept, or what fate awaits them after the end of the experiment [54]. To give an example of the information available in the quarterly publication of completed experiments, for an experiment conducted in 2023, one can see that a total of 294 mice were used in an experiment called “single unit recording” which was categorized as being the highest degree of severity (SD 3). The only further information that is published is that the experiment took place in the field of neurology for the purpose of basic research [55]. With this sparse information, it is at least questionable whether the Swiss government is meeting the requirements to inform the public sufficiently to assess the application of animal welfare legislation to animal experimentation, laboratory animals, and genetically modified animals [54]. Another issue that has been criticized at the political level is the lack of transparency regarding publicly funded animal experimentation, including animal facilities, as there is currently no legal requirement to make these figures publicly available [28].

Moreover, the Federal Council has not yet made use of its opportunity to publish further information on animal experimentation (cf. Art. 20a para. 2 AniWA). Moreover, the Council opted not to regulate the level of detail of the information published in accordance with Art. 20a para. 3 of AniWA, missing an opportunity to increase transparency in this matter [54]. Additionally, Art. 147 para. 3 of AniPO would oblige the FSVO to publish a periodic report in collaboration with the Federal Commission for Animal Experiments to inform the public about the development of animal welfare efforts in animal experimentation, laboratory animals, and genetically modified animals. To our knowledge, the FSVO has not yet published such a report.

In summary, the current public information falls short of enabling the public to form informed opinions [54]. Consequently, the public cannot properly take part in an objective and democratic debate on animal experimentation. We are therefore of the opinion that Switzerland, as a democratic state, does not reach a high standard of transparency (or even fulfill the Swiss state’s stated standard) and does not fulfill its duty to provide the public with a full account of animal experimentation (see above). In particular, there is a lack of information on the fate of the animals after the experiment, breeding/surplus animals, and funding, in our opinion. Accordingly, a “transparency turn” [56] in animal experimentation is long overdue. This would also be in line with the international tendency toward more transparency in animal experimentation as well as with Art. 147 para. 2 of AniPO, which obliges the FSVO to take international regulations and recommendations into account when compiling and publishing animal experimentation statistics. In the following section, we will take a closer look at the transparency regulations of the EU, which, while far from perfect, surpass Switzerland’s and could offer valuable insights for improvement.

## 4. Transparency Requirements in the European Union

EU Directive 2010/63/EU (subsequently Directive), which came into force on 22 September 2010, places great importance on transparency in the field of animal experimentation. Improving transparency is one of the main objectives of the Directive (Recital 4) [57,58], along with standardizing national regulations on animal experiments and increasing the welfare and overall level of protection of laboratory animals (Recitals 1, 2, 3, 6, 7, and 31).

Under the Directive, transparency is understood as “transparency to the general public of the performance of research establishments in terms of animal use and welfare” [58]. In the legislative process leading to the Directive, the right of access to information and its importance for a democratic society was particularly emphasized. The working paper accompanying the proposal for a Directive on the protection of laboratory animals stated: “If citizens are to exercise their democratic rights, and to make informed choices, they must have access to political, social, scientific and economic information” [59].

The Directive contains various elements designed to improve transparency. These include a project evaluation (Art. 36 and 38), inspections (Art. 34), and non-technical project summaries (NTS) (Art. 37 para. 1 lit. b and Art. 43), and reporting (Art. 54). The two elements of reporting and NTS, which are of particular interest here, will be explained below.

### 4.1. Non-Technical Project Summaries (NTS)

Non-technical project summaries (NTS) are considered a crucial instrument to make animal experimentation more transparent. According to Art. 43 para. 1 of the Directive, information on the objectives of the experiment, the expected harm and benefits, and the number and type of animals used must be recorded for each experiment in the form of an NTS. In order to ensure intellectual property rights and to protect confidential information and conflicting interests, the project summaries must be submitted anonymously (Art. 43 para. 1 of the Directive). The NTS are to be published by each Member State in accordance with Art. 43 para. 3 of the Directive as soon as the respective experiment has been authorized. They are then updated on an ongoing basis. However, it is up to the Member States to decide whether a project summary has to be adjusted retrospectively, i.e., after the experiment has been finalized [58]. In this context, the European Commission’s Directorate-General for the Environment has published guidance on how to carry out retrospective assessments of NTS, highlighting the potential of retrospective assessments to increase transparency and accountability, especially if their results are made public [60].

In addition to information on the objectives of the experiment and the application of the 3Rs principle (replace, reduce, refine), the fate of animals after the experiment must be made transparent for each experiment. For example, researchers must state whether the animals will be reused at the end of the experiment, returned to the habitat or husbandry system, housed privately, or euthanized [61].

In the EU Commission’s report on the review of the Directive, the Member States were asked whether the NTS had led to an improvement in transparency in the area of animal experimentation. Seventeen of the (at that time) 28 Member States answered in the affirmative, ten Member States could not (yet) give an assessment, and only one Member State denied an improvement in transparency [57]. However, there was also criticism from various animal welfare organizations. They criticized, for example, that the time to publish the summaries was too long and that the experiments were not reported in sufficient detail. The EU therefore recommends that scientists should receive better training concerning NTS and that NTS should be checked for accuracy before publication [57]. Furthermore, since 2021, Member States have had to comply with a deadline of six months for the publication of summaries (cf. Regulation (EU) 2019/1010). The NTS are published on an ongoing basis on the ALURES Non-Technical Summary EU Database, which is managed by the EU Commission [62].

### 4.2. Public Statistics

Regarding public statistics, Art. 54 para. 2 of the Directive stipulates that Member States must provide the public with annual statistics on the use of animals in experiments. Member States had to publish the statistics for the first time in 2015 (Art. 54 para. 2 of the EU Directive). In April 2020, the Commission adopted specific reporting formats in an implementing decision to standardize the Member States’ animal experimentation statistics (Commission Implementing Decision 2020/569). According to the implementing decision, the yearly statistics must include, for example, the origins of laboratory animals, the animals’ genetic status (i.e., whether or not the animals have been genetically modified), the degree of severity of harm, and the specific intended use of laboratory animals (see Annex III of the Commission Implementing Decision 2020/569).

In contrast with Switzerland, the EU Member States must also inform the Commission on the number of animals killed but not used in experiments (i.e., surplus animals). The Commission publishes the figures of all Member States on the Statistical Database ALURES [63]. Additionally, the most important figures are summarized in a report to assess the implementation of the EU Directive in the Member States. The first report, which was published in 2020, showed a total of 12.6 million surplus laboratory animals for 2017 alone [57]. According to the EU, surplus laboratory animals include all animals that “for one reason or another were not used or were unsuitable for scientific purposes” [57]. As the killing of animals for tissue or organ removal does not fall under the definition of animal experimentation in the EU (Art. 3 para. 1 EU Directive), these animals fall into the category of surplus animals as well [57]. However, the number of surplus animals is not part of the annual statistics, but the more general information within the meaning of Art. 54 para. 1 of the Directive and is therefore only to be submitted every five years (cf. Commission Implementing Decision 2020/569).

Given the above, it can be concluded that Switzerland clearly remains behind the EU Directive with regard to public information in the area of animal experiments, both in terms of NTS and reporting. This is despite the fact that Swiss institutions and politicians often claim that Swiss law provides animals with the best protections in the world, using a fallacy that has been called the “ranking argument” [64].

### 4.3. The German Case

Some Member States, such as Germany, go beyond the Directive in certain aspects of transparency. For example, the German Centre for the Protection of Laboratory Animals (Bf3R) launched the AnimalStudyRegistry database [65] in 2019. There, researchers can register and publish their experiments as early as the planning phase. However, such pre-registration has so far been voluntary. AnimalStudyRegistry aims to ensure that researchers also publish findings that do not lead to the desired results. Among other things, the initiative is intended to avoid duplication of animal experiments and aims to make the planning of experiments more thoughtful and targeted [66].

In addition, Germany has published the number of surplus laboratory animals annually with animal experimentation statistics since 2021 (Art. 1 para. 1a lit. b VersTierMEldV). Again in contrast with Switzerland, animals killed for scientific purposes are also listed separately in the annual statistics (Art. 1 para. 1a lit. a VersTierMEldV). This category includes animals that were killed to use organs or tissue for scientific purposes. In the case of animals used for scientific purposes, a distinction is also made between animals used for the first time and animals used again in an experiment [67].

The publication of the number of surplus animals has led to a remarkable reaction in Germany. Based on published figures, two German animal welfare organizations filed criminal charges against 15 laboratories in June 2021. The criminal charges were filed on the grounds that there was no reasonable justification for the killing of surplus animals [68]. The background to this was a judgment by the German Federal Administrative Court in 2019, dealing with the killing of surplus male chicks in the egg industry. The court ruled that the killing of male chicks could not be justified by a reasonable cause [69]. The killing of these chicks (i.e., of the species *Gallus gallus*) has been prohibited by law in Germany since 2022 (Art. 4c TierSchG).

Although the proceedings on surplus laboratory animals were all discontinued or not taken up, they did lead to a change in scientific establishments. Many research institutions reduced the killing of surplus animals, stopped it altogether (for at least some time), or invested more resources in the development of alternative methods [70], leading to a decrease of 33 percent in the number of surplus/breeding animals from 2021 to 2022 [71]. As a result, the German Federal Institute for Risk Assessment (BfR) also looked into the killing of surplus laboratory animals [71,72]. It concluded that the killing of these animals should be carefully considered on a case-by-case basis. In addition, the “further use of the animals for other scientific questions” should be carefully scrutinized. Alternative options, such as keeping the animals in laboratory animal facilities or private institutions, should also be considered [73].

These developments show how important transparency is in the field of animal experimentation. By publishing the numbers of breeding and surplus animals, this previously non-disclosed aspect of animal experimentation became visible to the public and enabled public action [74].

## 5. Toward More Transparency in Public Information Under Swiss Law

We have now discussed the state of transparency in the Swiss, European, and German contexts. While progress has been made in recent decades, we cannot consider these states of transparency in public information as fully compliant with the *agent accountability view*. In other words, in each of these contexts, the public does not seem to have all the information necessary to assess the government’s animal experimentation activity done on its behalf in a way that enables satisfactory informed consent, legitimate state action, or political participation. Focusing on the Swiss context again, we said above that to progress toward more transparency in public information about animal experimentation one crucial question must be answered: What information on animal experimentation should be part of the government’s full account to the public?

Another important issue that may arise is the accessibility and comprehensibility of information published by the government, which as we said is a necessary condition for transparency. We mentioned above that Swiss law does not specify exactly how and through which channels the public should be informed about animal experimentation. As explained, transparency requires comprehensibility. In this sense, all of our recommendations implicitly assume that the information should always be published in a comprehensible and easily accessible manner to meet transparency requirements. In this sense, the question could also be raised whether the proposed web-based statistics, figures, and reports are sufficient to ensure transparency, or whether other forms of communication, such as photos or videos, occasional live streams of laboratories, or laboratory tours for the public, would be a better approach. However, such efforts must be made in collaboration with the institutions themselves. The aforementioned STAAR-Initiative has made an effort to encourage its signatories to organize “public events such as debates, open days, etc.” [6]. While such initiatives aiming at improving the “how” and “where” of transparent communication about animal experimentation are necessary, this article focuses on “what” information should be disclosed, and it is outside this article’s scope to assess these efforts. We nonetheless consider such efforts essential, in particular because animal experimentation is a highly technical field. As we said above, only well-informed citizens can form their own opinions and take an active part in the decision-making process. In this sense, increasing the amount of information without ensuring its comprehensibility would miss the target. While completely closing the gap of scientific knowledge between science and the public might be an impossible task, we do not argue here that decisions in science should merely be determined by public opinion. We believe that transparency efforts contribute to the aim of having well-informed citizens able to take an active part in these decisions.

We argued that all of the ethically, politically, and legally relevant facts that matter to citizens should be disclosed if we are to allow a public assessment of the government’s animal experimentation activity. To illustrate what we mean by ethically, politically, and legally relevant facts, let us consider, for example, the killing of animals in science. Under Swiss law today, (painless) death is not considered a harm and is therefore categorized as degree of severity 0. Some animal ethicists consider that animal death is a harm [53,75], while others do so depending on the cognitive faculties of the animal in question [76], but some do not [77]. The expert consensus in the Swiss scientific and legal context is in line with the last position, namely that (painless) animal death is *per se* in some sense ethically irrelevant (despite all creatures supposedly having dignity under the Swiss constitution). However, a full account by the government must consider all of these views and include what citizens consider ethically and politically relevant. Public demonstrations and political actions in Europe have shown that citizens consider the killing of animals as ethically and politically relevant [78,79]. In this sense, even if it could be considered legally irrelevant, the killing of animals is an ethically and politically relevant fact that must be disclosed in a full account, if the citizens are to assess the government’s activity. It could be argued that higher transparency expectations are attached to animal experimentation funded by public money as compared to private money, given the interest of citizens to be informed about the use of tax money. Citizens have an interest in considering the costs of animal experimentation activities to assess whether public money is being spent in the right way [32]. While such evaluation does often have ethical implications, it must mainly be made transparent as a matter of law and politics.

Having these considerations in mind, we will now suggest how to move toward more transparency in public information about animal experimentation under Swiss law, arguing for the inclusion of further ethically, politically, and legally relevant facts for a public assessment of the Swiss government’s animal experimentation activity done on the public’s behalf. When information is clearly relevant in ethical, political, or legal terms, or two or all three, for the purpose of clarity we will not always specify which applies. To provide a better overview, the recommendations below are also summarized in the following table (Table 1):

### 5.1. In Experimentation

As we have seen, current Swiss law allows the public to know the following information about animal experimentation in Switzerland, based on the licensing and reporting obligations in animal experimentation:

− Numbers of animals used in experiments
− Species of the animals used in experiments
− Degrees of severity of harm experienced by the animals used in experiments
− Objective and subject area of the experiment
− Title of a research project

The number of animals used in experiments is relevant information for the public, not only based on the law but also on the public interest in each individual sentient animal. The same goes for the degree of severity of harm experienced by these animals, as their sentience means that they can experience pain and pleasure and that all welfare considerations in Swiss law apply to them. From a social or cultural perspective, it is understandable that the public cares about the type of species used in experiments as they have different attitudes about and relationships with different species [80]. One example of that is still present in Swiss law today, where it is stated that experiments should be performed on animals that are “lower in the evolutionary scale” (Art. 20 para. 2 AniWA) when that is possible. From a biological perspective, no such scale exists, and the concept reflects a rather anthropocentric perspective [81]. Nonetheless, the legal concept of “evolutionary scale” suggests that transparency about the species used is relevant. The objective and subject area of the experiment are also clearly relevant, since, as we have seen, the justification of animal experimentation depends on a harm-benefit analysis and the public has different attitudes about research objectives and fields. Finally, the title of a research project is somewhat relevant for pragmatic reasons (e.g., communication or information seeking about a particular project), but seems to be the least important aspect on the list.

There is, however, more ethically, politically, or legally relevant information about animal experimentation that should be transparent in the government’s full account. Below, we will argue for the inclusion of the following:

− Fate of the animals at the end of the experiment
− Costs and funding details of animal experimentation
− A comprehensible presentation of the harm-benefit analysis.

#### 5.1.1. Fate of the Animals

Not only is the killing of animals (even if painless) a highly debated issue in animal ethics, it also clearly matters to the public. While the killing of animals used as pets has been relevant in public opinion for a long time, it has now become crucial in other types of animal use [4]. The unnecessary killing of male chicks in the egg industry, like that of surplus animals in zoos and in the breeding of laboratory animals, led to extensive debates in the literature and public discourse, as well as political actions [82,83,84,85]. In its most advanced forms, it led to a ban on the killing of male chicks, as well as criminal charges concerning surplus laboratory animals, in Germany (see above). Currently, the public cannot know how many animals are euthanized or killed [we distinguish here between euthanasia, the mercy killing of animals in their own interests to avoid unbearable harm, and killing, which can be painless but is not in the animals’ own interests], during or after experiments in Switzerland. While it is no secret that the vast majority of the animals used in experiments are killed [50], three other fates are possible. Some animals are rehomed in private homes or dedicated facilities. Rehoming gained interest in the last years, leading to projects facilitating the process (see for example the rehoming-project of the Swiss Animal Protection Organization STS and the University of Zurich, which managed to rehome hundreds of mice, rats, rabbits and dogs) [51]. Other animals, especially smaller rodents, are sometimes reused in a different experiment or used as feed [52], “optimizing” their sacrifice, in a sense. But these two fates are very rare, not only because a majority of laboratory animals do not have a profile allowing them to be placed in a private home or facility, e.g., genetically modified animals (Art. 6 para. 3 of the Federal Act on Non-Human Gene Technology (GTA), or to be of use for a different research project (cf. Art. 135 para. 8 AniPO.), but also because initiatives to facilitate these options are limited and the economical, logistical, and systematic incentives tend to be on the side of killing. Even more rare (due to logistic and economic reasons) is the third possibility, which would be to keep the animals in the research institution or animal facility until they die naturally of old age or disease. Moreover, the fact that approved killing methods, like death itself, are considered as degree of severity 0 under Swiss law makes death a “hidden cost”. Indeed, in 2022 the account of the government shows that 219,241 animals were “used” in a degree of severity 0 experiments. That represents 37.4% of all animals used, making the degree of severity 0 the most common and potentially leading the public to believe that most animals are “used” without experiencing any type of harm. More research should be done to investigate how clearly the public understands the degrees of severity, but categorizing painful (even if slightly) killings in a degree of severity numbered as 0 and labeled as “no harm” is not the most transparent approach, at least in terms of comprehensibility.

The question then remains: what proportion of the 585,991 animals used in experiments in Switzerland in 2022 have been euthanized, killed, reused, rehomed, or died naturally? When the public learns that 585,991 animals have been “used”, this is somewhat misleading. Indeed, the term “use” does not entail a particular fate. The public’s opinion on animal experimentation could be different (even if slightly) if transparent numbers about their fate were accessible, and it would allow for the provision of public information closer to a full account. One could argue that how animals are killed is also of interest. The degree of severity of harm reported by researchers relates to what happens before death. The legally approved killing methods do not cause a change in that degree of severity of harm. But the ways in which animals are killed in or after an experiment are diverse, and while some could be considered as almost painless, others could be considered stressful, and some as not even respecting animal dignity. For example, smaller rodents are still often killed using CO_2_, although various studies show that this method causes pain and stress for the animals [54]. Taking all of this into consideration, we therefore recommend Swiss law to require the provision of public information about the number of animals euthanized or killed during or after experiments, as well as reused, rehomed, or kept until natural death. This could be accomplished without difficulty, as researchers are required to report the fate of animals after an experiment anyway (Art. 145 para. 2 AniPO; Art. 30 para. 1 lit. e AEO.), so these data already exist. It is not entirely clear why some of the information covered by the reporting obligation, such as the final severity of harm, is publicly available, while other information, such as the fate of the animals, is not, despite its ethical relevance. We therefore argue that the fate of animals should be included in the quarterly report according to Art. 145a of AniPO, where it should be stated for each experiment how many animals were euthanized, killed, reused, rehomed, or died naturally at the end of the experiment. In addition, a justification for the intended fate of the animals should be required, as is the case for NTS, and the total numbers should be broken down in the annual statistics to provide a complete account.

#### 5.1.2. Funding of Animal Experimentation

While annual statistics show whether an experiment is conducted by a state institution, such as universities or public hospitals, or by a private institution, they do not differentiate between the sources of funding (public or private) [28]. Although the Swiss National Science Foundation (SNSF), which handles the majority of federal research funds, publishes its annual investments in research, the amount that is effectively used for animal experimentation can only be estimated. It is also not clear how much of the federal funding goes into research on animal-free methods [86]. As a consequence, the public is often unaware of the impact of their taxes on animals used in research. But as stated above, a large proportion of animal testing is publicly funded, and the public benefits (at least to some extent) from that research [32,87]. It is only logical that it is in the public’s best interest to fund research with the highest benefits and the least amount of suffering for animals possible. Therefore, if the public is to assess the use of state funds by the government in research activities done on its behalf, it must be able to compare, for example, animal experimentation to other types of approaches in terms of funding, harms, and benefits. However, with the current information, the public is not able to judge whether public funds for research are being distributed in its best interest. It is especially important for the public to know the total amount of state-funded animal experimentation in comparison to non-animal methods for at least three reasons: First, the evolution of state investments can serve as a metric to monitor the progress of non-animal methods. Indeed, the Swiss government must promote methods that replace animal experiments (Art. 22 para. 2 AniWA.), for example, by creating a competence center (i.e., Swiss 3RCC). It is therefore relevant for the public to know if progress toward this shift is satisfactory and whether the government is fulfilling its obligation to promote replacement. Second, the law states that animal experimentation can only be conducted when there are no adequate alternative methods, and the public should be able to assess the application of the law through public information on animal experimentation (Art. 137 para. 2 AniPO). Third, some evidence suggests that non-animal methods may offer certain advantages over traditional animal studies, particularly regarding human relevance and reliability in specific research areas [88,89].

Due to the lack of public information in this area, the public cannot conduct a conclusive assessment of the state’s funding for animal experimentation. In the interest of government legitimacy, public awareness, and scrutiny, moving toward more transparency in this regard is therefore essential. This could be achieved by including the source of funding (i.e., public or private) and the amount for each experiment in the quarterly statistics published under Art. 20a of AniWA. To ensure that the information is easily accessible and understandable, this could be done in the form of NTS for each experiment. Although the EU does not currently require information on funding in NTS, this could easily be included. Moreover, the annual report accompanying the statistics should disclose how much research using animals the state funded in comparison to research on animal-free methods.

#### 5.1.3. Harm-Benefit Analysis

As stated, the public in Switzerland is informed about the objective and subject area of an animal experiment. This is crucial because, while the concept of harm-benefit analysis has been challenged in terms of its limitations [90], the justification of animal experimentation in the Swiss context does depend on it. However, merely knowing about the objective and subject area is not enough for the public to assess the ethical justification of an experiment. Indeed, knowing that an experiment in the field of “neurology” has as its objective “basic research in biology” is still quite vague. The same holds true for the degrees of severity of harm. Although these are described in more detail in various FSVO guidelines [91], they are still rather abstract and provide only a rough description of different harms. The public cannot get a picture of what happens to individual animals in the experiment, particularly if they are not familiar with animal experimentation [54].

An interesting case to illustrate this comes from the Zurich Administrative Court. In its 24 November 2022 ruling, the Zurich Administrative Court addressed an application for a severe animal experiment on zebra finches (severity grade 3). The experiment involved genetically modifying the birds’ brain cells to study birdsong and, as a long-term goal, to gain new knowledge about human language acquisition. Over 100 zebra finches would have undergone cranial surgery, been connected to a cable, and kept in isolation for extended periods, with most being euthanized afterward. The Veterinary Office of the Canton Zurich, as the licensing authority, approved the experiment with conditions at the request of the Animal Experimentation Committee. Subsequently, three members of the Committee appealed to the Health Directorate, which rejected the appeal [92]. The case was finally brought to the Zurich Administrative Court, which did not grant permission for the experiment because the expected benefit did not outweigh the severe harm to be inflicted on the zebra finches (cf. Art. 19 para. 4 AniWA). The decision is final and uncontested [92]. Such cases are clearly of public interest, and the commission members opposed to the approval of the experiments played an important role in this case. While it is difficult to inform the public of all of the relevant details to assess such a case before it goes to court, merely knowing that it concerns, for example, basic research in neurology, is not good enough.

With regard to the purpose of the experiment, a more comprehensible account of the knowledge and benefit that the experiment aims at is needed. In the case presented above, researchers wanted to find out which mechanisms in a bird’s brain are responsible for birdsong, and they intended to use that knowledge to help understand human communication [92]. To do this, the researchers planned to subject the birds to multiple cranial surgeries and tether them for several days in a row. After the experiment, most of the birds would be euthanized. The experiment was categorized at degree of severity 3 [92]. If this case had not gone to court, the public would never have known the expected benefits or the exact harms of this experiment, only that it was a basic research experiment in the field of neurology within degree of severity 3.

Knowing the expected benefits, as well as the harms of the experiment and the number of animals used, brings the public closer to a realistic assessment of the ethical justification of such experiments (cf. Art. 19 para. 4 AniWA). Therefore, we recommend that public information should also include comprehensible information about the purpose of the experiment, clarifying the rationale and expected benefits. To avoid selective openness, i.e., the disclosure of only positive information by researchers [93], and move closer to a full account, public information needs to provide a more detailed description of the harms (beyond the rather abstract degrees of severity of harm), the benefits, and the purpose of an individual experiment. Again, here, NTS would be a good way to inform the public. However, Switzerland should go further than the EU in terms of the content of NTS. As previously stated, NTS state, among other things, the expected benefits and harms to the animals, and details of procedures and manipulations. Nonetheless, no proper harm-benefit analysis is required or presented. If Switzerland were to introduce such NTS, the standard NTS should include a proper harm-benefit analysis. Any approved experiment in Switzerland must describe such a harm-benefit analysis as part of the application procedure for a license to perform an animal experiment. In this context, it can be argued that a further step toward transparency would be to make the original evaluation reports of the approval procedure available, in addition to an NTS.

A model for the implementation of NTS in Switzerland could be the study portal for clinical trials involving human subjects [94]. However, such a portal would have to be adapted to the particularities of animal experimentation. A good way to do this would be to favor an interactive process during the establishing phase, including the feedback of members of the public regarding aspects such as the wording, the relevance of the content, and more.

### 5.2. In Animal Facilities

When it comes to Swiss laboratory animal facilities, current Swiss law allows the public to access the following information, based on facilities’ license and reporting obligations:

− Number of animals born in facilities
− Number of animals imported from abroad

Here again, because of the law and public interest in the use of sentient animals, the number of animals in the custody of the facility, either from birth or after importation, is relevant information. In this sense, several relevant categories of information are missing in our view, if the government is to fulfill its duty to give a full account of animal experimentation activities done on behalf of the public. Below, we suggest the inclusion in the Swiss government’s full account about animal experimentation of the following:

− Number of breeding/surplus animals
− Fate of breeding/surplus animals
− Harms experienced by animals in facilities
− Funding of animal facilities

As explained above, we use the term “breeding/surplus animals” to refer to all animals involved in animal experimentation but not used in actual experiments.

#### 5.2.1. Number of Breeding/Surplus Animals

As already stated, one could try to get a sense of how many breeding/surplus animals exist in Switzerland by comparing the reported number of animals born or imported into Swiss animal facilities to the reported number of animals used in experiments. However, any such estimation would not be accurate for multiple reasons: First, some animals born or imported into animal facilities spend multiple years there and are only included in the statistics for the first year. Second, some animals used in experiments are reused for multiple experiments. Third, not all animals go through a licensed facility before being used in animal experiments. For example, animals that are not imported from abroad or bred in the facility, i.e., livestock from private farms, or pets used in an experiment, are not included in the figures on animal facilities, since existing domestic, wild, and pet animal facilities in which laboratory animals are kept occasionally or temporarily do not require a license according to Art. 122 para. 6 AniPO.

The non-disclosure of breeding/surplus animals is salient when such use is merely to bring animals into existence and kill them soon after, a use that conflicts with the notion of dignity in Swiss law which, for example, excludes excessive instrumentalization (Art. 3 lit. a AniWA.). For these reasons, the transparent disclosure of the exact number of breeding/surplus animals, including the number of breeding and the number of surplus animals in these, should be mandated by Swiss law. At present, no data are collected on breeding/surplus animals. However, with the ongoing revision of AniPO and the AEO, this is expected to change. The draft of Art. 29 para. 1 lit. d proposes that the number of surplus animals as well as the number of animals used for breeding should be reported by laboratory facilities. While we commend this proposed step toward more transparency, it is important to ensure that this information is publicly disclosed once it is included in the reporting duty of the facilities.

#### 5.2.2. Fate of Breeding/Surplus Animals

The fate of breeding/surplus animals is either to die in a facility or to be rehomed. In principle, a breeding animal can also die naturally of old age or disease. However, from an economic and logistical point of view, this is rare. Breeding/surplus animals killed by humans, not in their best interests but for economic and logistical reasons, pose a significant moral problem which (as already shown) is of great interest to the public. Breeding/surplus animals that are rehomed, whether in a dedicated facility or private homes ensuring conditions compliant with their welfare, while representing a minor proportion, represent a better solution from a moral perspective. Because these outcomes are morally significant to the public, Swiss law should require transparency about the fate of breeding/surplus animals. In this regard, too, the ongoing revision is intended to increase transparency. In addition to the number of surplus animals, the draft requires the reporting of the number of rehomed animals, the number of animals that died spontaneously, and the number of animals with an unknown fate. As stated in the explanatory report on the consultation process, the fate of animals is already documented by facilities as part of ‘inventory control’ and would have to be reported. Again, we hope that these data will be made available to the public as soon as it is collected.

#### 5.2.3. Harms Experienced by Animals in Facilities

The lack of data on the details of the living (and dying) conditions of animals in facilities conveys the idea, or at least can be read as implying, that their lives (and deaths) involve no harm whatsoever. However, the reality of breeding and husbandry in animal experimentation does not reflect that. The literature suggests that breeding and husbandry conditions in animal experimentation may impose harm on animals due to social separation, isolation, anxiety (e.g., transportation, handling), small cages, and death [87,95,96]. In some respects, animal facilities are more strictly regulated than, for example, the housing of animals in private homes, but in other ways, they have more discretion and can even deviate from the legal minimum requirements, if authorized and necessary for the purpose of research (Art. 113 AniPO). However, harm may occur even if a facility complies with Swiss law on the husbandry of animals, and it matters for the same reasons as harm occurring in experiments. For example, a comparison of the legally prescribed minimum space for laboratory animals with that for pets of the same species shows that laboratory animals are often granted very little space. For example, while the minimally required floor area for two small pet mice is 1800 square centimeters, it is only 330 square centimeters for two laboratory mice that are not used for breeding (Annex 1 and 3 AniPO). Moreover, in practice, social animals are still sometimes kept in solitary confinement, despite the known negative effects on their wellbeing, such as behavioral disorders or organic diseases [54]. With regard to the breeding conditions of laboratory animals, it can only be speculated that these are also associated with harm to the animals, as there are little to no data on this. Moreover, research shows that laboratory housing increases morbidity and mortality in some species, including rodents [97]. Animal facilities have to monitor the health, welfare, and hygiene status of the animals, but this requirement is not subject to reporting. There are also no clear indicators of how to measure and classify harm in laboratories, as there are for animals used in experiments.

Genetically modified animals are a special case, because they are attributed a degree of severity of harm whether they are used in experiments or not (Art. 124 AniPO; Art. 25 AEO). Their wellbeing has to be documented regularly and it must be reported if the animal experiences harm (see above). From an ethical perspective, however, it is not clear how such a distinction can be made between the harm experienced by genetically modified animals and other types of animals in facilities. One could therefore argue that Swiss law should be changed in such a way that animal facilities would have to document and report harm to animals that have not been genetically modified as well. Whether the degrees of severity of harm are fit for such a purpose or other measures are needed, however, is a question that falls outside the scope of this article. For now, let us say that at the very least the public should be informed whether the animals’ husbandry conditions were in line with the law or deviated from the minimum standards following Art. 113 of AniPO. That the public is interested in husbandry conditions has been shown in a survey conducted among Swiss citizens in 2007, in which 75% of the respondents stated that they would be in favor of an improvement of husbandry conditions in laboratory animal facilities [98]. To achieve more transparency in this regard, the facilities would have to start documenting and reporting the necessary data on their husbandry conditions and in the best case, go one step further and report whether animals that are not genetically modified experience harm because of the conditions in the facilities. This could be included in the mandatory ‘inventory control’ (Art. 143 AniPO) as is the case for genetically modified animals.

Furthermore, with regard to genetically modified animals, Art. 147 of AniPO states that the statistics should contain the necessary information to assess the application of animal experimentation legislation in the area of genetically modified animals. So far, however, the only thing the public knows about genetically modified animals is how many were used in experiments and to what degree of severity of harm they were subjected in an experiment. This is not enough information for the public to adequately assess the application of the legislation in this area. Therefore, at least the data that are already collected (i.e., the harm resulting from genetic modification of animals) should be made available to the public. To ensure that conflicting interests, such as the protection of business or manufacturing secrets or the privacy and security of researchers and research institutions, are protected, the data should be collected and published anonymously.

#### 5.2.4. Funding of Animal Facilities

As noted above, there is currently a lack of transparency in publicly funded animal experimentation, resulting in a lack of public awareness and scrutiny. This is even more true for the funding of animal facilities, where even less information is available. As we have seen, even when it is not required by law, the SNSF discloses information about the funding of animal experimentation, including comparisons of funding for other types of research. Such information is not available for the public funding of animal facilities in Switzerland. As we have argued throughout this article, a clear distinction between animal facilities and actual experiments does not always hold when we consider ethical, legal, or political aspects. This is certainly true when it comes to the public interest in having a transparent, comprehensible view of tax money invested in the use of animals in this field.

When discussing the funding of animal experimentation above, we emphasized the need to be able to compare it to progress in funding innovative alternative methods. Transparency with regard to the funding of animal facilities can serve a similar purpose, as it would allow a comparison with the progress in funding alternative infrastructures (e.g., laboratories dedicated to non-animal methods) and their resources. While Switzerland, unlike the European Union (Recital 11 EU Directive), does not present the full replacement of animal experimentation as one of its goals for the future, the attitude of a significant part of its citizens suggests that this should be the aim [99]. In any case, monitoring the use of taxpayers’ money in these diverse technologies is necessary, if only for the public to assess whether these resources should be invested in different approaches that are more effective, more ethical, or present an advantage regarding any other value.

Here again, because of the lack of public information, a conclusive assessment of the state funding in animal experimentation (viewed more broadly) cannot be conducted by the public. For the same reasons as stated above (i.e., the interest of government legitimacy, public awareness, and scrutiny) moving toward more transparency is necessary [28]. This could be achieved by disclosing the public funding of Swiss animal facilities dedicated to animal experimentation. Since these figures are already collected by Animex-ch for licensed facilities [54], it would be a simple step to make them available to the public.

## 6. Conclusions

In this article, we have argued that there is an ethically and legally problematic lack of transparency in Switzerland when it comes to public information about animal experimentation, considering that the government has a duty to provide a full account of its use of animals in experimentation on behalf of the public. While efforts to tackle this issue have risen in the scientific community, these mostly reflect the legal requirements already in force. If Switzerland is to move toward more transparency in public information on animal experimentation, we argue that an update of the legal requirements is needed. In this sense, we provide recommendations for Swiss law to move toward more transparency in public information on seven aspects: (1) the fate of the animals at the end of the experiment; (2) the funding of animal experimentation; (3) the harm-benefit analysis; (4) the number of breeding/surplus animals; (5) the fate of breeding/surplus animals; (6) the harms experienced by animals in facilities; (7) the funding of animal facilities. To do so, we defined our account of transparency, presented current Swiss law on the issue, and compared it to the general European and, in particular, the German context.

While our seven recommendations can be categorized as either concerning actual experiments or animal facilities, transparent public information would need to offer a complete picture of the use of animals in research. Indeed, the existence of breeding/surplus animals is necessary for animal experimentation and must therefore be presented as such. In this sense, we suggested instruments through which this new information could be communicated to the public, some of which are already in place, like the annual and quarterly reports, and others that should be implemented, such as non-technical summaries and a study portal similar to that for clinical trials involving human subjects.

These improvements in transparency could be looked at with caution, even more so in a country known for regularly bringing the debate about animal experimentation into the public arena. However, different stakeholders will have different reasons for why animal experimentation should be more transparent. While researchers may argue that transparency is important to counteract potential damage to the public image and to increase public trust in the conduct of animal experimentation, animal welfare advocates may argue that transparency is important to raise public awareness, stimulate public debate, ultimately improve animal welfare standards, and accelerate the use of alternative methods. There are diverse interpretations and rationales surrounding transparency in animal experimentation, but there is a broad consensus on its importance. Although transparency in animal experimentation can and should include more than accountability (e.g., involvement of the public in the decision-making, collaborations between the media and the scientific community, and access to facilities by the public), this article focused on public information and the particular duty of the government to provide a full account of its use of animals in experimentation on behalf of the public. In this sense, our recommendations are intended as a move toward more transparency when it comes to the critical question “What information should be disclosed to the public?”.

## Figures and Tables

**Table 1:** Recommendations for greater transparency in public information about animal experimentation in Switzerland.

Information	Description	Public Information
Fate of the animals	An account of the fate (i.e., killing, euthanasia, natural death, rehoming, or reuse) of animals used in experiments.	Annual report; Quarterly report; Non-technical project summary.
Funding of animal experimentation	An account of the amounts of state funds invested in animal experiments.	Annual report; Quarterly report; Non-technical project summary.
Harm-benefit analysis	A comprehensible account of the purpose of the experiment (expected benefits) as well as the harm to the animals.	Non-technical project summary; Study portal (like that for clinical trials involving human subjects).
Number of breeding/surplus animals	An account of the exact number of breeding and surplus animals.	Annual report.
Fate of breeding/surplus animals	An account of the fate of breeding/surplus animals (i.e., killing, euthanasia, natural death, rehoming).	Annual report.
Harms in animal facilities	An account of deviations from the minimum legal standards for husbandry conditions.	Inventory control; Annual report.
Funding of animal facilities	An account of the amounts of state funds invested in animal facilities.	Annual report.

## Data Availability

Data are contained within the article.
